# Mass casualty decontamination following a chemical incident: evaluating improvised and interim decontamination protocols in a controlled cross-over volunteer study

**DOI:** 10.1136/emermed-2024-214221

**Published:** 2024-12-04

**Authors:** Louise Davidson, Felicity Southworth, Natalie Williams, Thomas James, Emily Orchard, Tim Marczylo, Samuel Collins, Richard Amlôt

**Affiliations:** 1 Behavioural Science and Insights Unit, UK Health Security Agency, Porton, UK; 2 Radiation, Chemical, Climate, and Environmental Hazards, UK Health Security Agency South East, Chilton, UK; 3 Global Operations, UK Health Security Agency, London, UK

**Keywords:** effectiveness, disaster planning, emergency responders, major incident, CBRN

## Abstract

**Background:**

On-scene improvised and interim decontamination protocols in the Initial Operational Response to chemical incidents aim for rapid intervention to minimise injury before specialist capabilities arrive. This study examines the effectiveness of UK improvised and interim protocols conducted in sequence.

**Method:**

A simulant with methyl salicylate (MeS) in vegetable oil and a fluorophore was applied to participants’ shoulders, arms and legs. Participants either received no decontamination or used one of four decontamination protocols: improvised dry, improvised wet, improvised dry followed by interim or improvised wet followed by interim. Remaining simulant on the skin was quantified using gas chromatography tandem mass spectrometry for MeS analysis and UV imaging for fluorophore detection. Additionally, urine samples were collected for 8 hours post application to analyse MeS levels.

**Results:**

Significantly less simulant was recovered from the skin post decontamination compared with no decontamination. There were no differences in the total simulant recovered across all decontamination conditions. However, significantly more simulant was recovered from the shoulder compared with the arm and leg. Variation in simulant recovery from different application areas was significantly higher in improvised-only conditions than in combined conditions. Decontamination did not affect the amount of MeS excreted in urine over 8 hours.

**Conclusion:**

This research supports current practice of starting decontamination as soon as possible after chemical exposure and highlights the importance of implementing interim decontamination following improvised decontamination.

**WHAT IS ALREADY KNOWN ON THIS TOPIC**

- Sequential improvised decontamination procedures remove more simulant than isolated decontamination procedures.

**WHAT THIS STUDY ADDS**

- Under experimental conditions with healthy participants and a simulant, this study found that interim decontamination following improvised methods more effectively decontaminate harder-to-reach areas of the body often missed during improvised decontamination.

**HOW THIS STUDY MIGHT AFFECT RESEARCH, PRACTICE OR POLICY**

- This study supports current UK emergency decontamination guidelines and recommends prompt use of interim decontamination following chemical exposure.

## Introduction

 Incidents involving the unexpected or uncontrolled release of hazardous chemicals create an exposure risk to the public and emergency decontamination may be required to reduce injury and prevent loss of life. Effective decontamination involves actions that reduce, remove, neutralise or inactivate contaminants,^1^ thereby preventing further uncontrolled spread. Due to the complexity of chemical incidents, responders must manage casualties and implement timely decontamination. Therefore, strong scientific evidence is essential to ensure timely and effective decontamination.

In recent years, research on emergency decontamination, including in vitro studies^2 3^ and human volunteer trials,^4^,^8^ has enhanced emergency responses to chemical incidents. In the UK, the focus has shifted from relying on Fire and Rescue Service (FRS) mass decontamination units, known as the Specialist Operational Response (SOR), which can require substantial time to set up, to a more rapidly deployable Initial Operational Response (IOR). This change, driven by new evidence on improvised methods,^7 9^ aims to quickly decontaminate casualties, minimising risks before specialist resources arrive.

UK guidance emphasises that the first 15 min after exposure to a hazardous substance are critical for life-saving actions.^10^ IOR provides the emergency services with guidance for what immediate steps should be taken when an incident is reported, as well as advising the public on what they can do. IOR begins with the initial report, ensuring prompt advice and response, prioritising life-saving efforts regardless of an incident’s cause. Specialist resources can then transition from IOR to a more thorough SOR as needed.^10^

IOR strategies emphasise three ‘remove’ principles^10^: first, remove casualties from the contamination source; second, remove potentially contaminated clothing as disrobing greatly reduces exposure effects; and third, remove the substance using improvised decontamination methods. This study focuses on the final ‘remove’ strategy.

IOR guidance suggests using improvised dry decontamination as the primary method for non-caustic chemical exposure by blotting and rubbing affected areas with dry materials, such as tissue or cloth.^11^ For suspected caustic agents, improvised wet decontamination is recommended, using water from any available source to dilute and flush the chemical agent away from the body.^11^ This involves the rinse–wipe–rinse procedure: rinse with clean water, wipe with water and detergent, then rinse again. Interim decontamination typically follows improvised methods using standard FRS equipment, often forming an improvised shower corridor with hoses and ladders.

It is generally assumed that decontamination is more effective when procedures are applied sequentially. For example, a human volunteer trial showed that combining improvised dry and wet decontamination methods was more effective at removing a chemical simulant than using them separately.^7^ However, the study also found that improvised methods were less effective at removing the chemical simulant for hard-to-reach areas, such as the back of the shoulders, compared with easier-to-reach areas, such as arms and legs. Interim decontamination may better address hard-to-reach areas by using a full-body shower instead of relying on casualties to wipe or pour water on specific body parts. However, the added benefits of interim decontamination after improvised methods, particularly for hard-to-reach areas, remain unexplored. Thus, this study aims to assess the effectiveness of improvised and interim decontamination protocols, individually and combined.

It was hypothesised that each decontamination protocol would remove more simulant from the skin and reduce levels of urinary methyl salicylate (MeS) excreted by volunteers than a no decontamination control. Furthermore, it was expected that more simulant would be removed from the skin when improvised and interim decontamination procedures were combined, compared with when conducted separately. Finally, it was expected that more simulant would be removed from hard-to-reach areas of the body (eg, shoulders/upper back) when interim decontamination was conducted following improvised decontamination than improvised alone.

## Methods

12 adults (5 women, 7 men, ages 25–50 years) completed a controlled, cross-over study between May and July 2018 at a Public Health England site near Salisbury, UK. Participants were recruited from Public Health England staff and neighbouring sites. They completed five decontamination conditions in a randomised order. Study sessions were separated by a minimum of 3 days to allow systemic MeS levels to return to baseline.

Five decontamination conditions were tested: no decontamination control (A), improvised dry (B), improvised wet (C), improvised dry and interim in sequence (D) and improvised wet and interim in sequence (E). Decontamination conditions were conducted according to the protocols outlined in table 1. Descriptive statistics of decontamination condition characteristic variables are presented in table 2.

**Table 1:** Decontamination conditions

Decontamination condition	Protocol description
Control (A)	No decontamination. Participants were asked to move to the different predefined decontamination areas to replicate the movements of participants in the decontamination conditions.
Improvised dry (B)	This condition followed the IOR dry decontamination procedure^1^ with the following modifications: pieces of white roll (Hygiene Rolls White 2-Ply 25 cm × 25 cm, Tower Supplies, Poole, UK) were individually folded in half two times. Participants were instructed to use one piece of white roll at a time and to blot and rub their skin working down the body from the shoulders. Participants were reminded not to come back up the body using the same piece of white roll. Participants were given a total of 3 min but were instructed to stop decontaminating when they felt they had finished. Participants were free to use as many pieces of white roll as they felt they required.
Improvised wet (C)	This condition followed the IOR rinse–wipe–rinse procedure.^1^ Three buckets containing 5 L of ambient temperature water were provided, with one bucket containing 0.5% detergent solution (Fairy Liquid, Procter and Gamble, UK). Decontamination lasted a total of 3 min. In three stages lasting 1 min each, participants were instructed to: Rinse the body using clean water and a 1 L jug provided. Wipe themselves using a sponge and the detergent solution, in a downward motion from the shoulders, not returning up the body. Rinse for a final time using clean water and the 1 L jug.
Improvised dry and interim in sequence (D)	This condition followed the IOR improvised dry decontamination procedure as outlined above and an interim decontamination protocol designed by the research team and agreed by UK first responders. A ‘ladder-pipe’ shower system was set up by trained fire service responders using four hose reels and branches, which created two shower positions. Decontamination lasted 90 s in total across two stages, with each stage lasting 45 s. Participants were instructed to: Walk to the first shower position and actively wash themselves from the shoulders downwards using their hands. Move to the second shower position and complete a slow 360° turn with their arms held to the side at shoulder height and rinse their hands in the water once the turn was complete.
Improvised wet and interim in sequence (E)	This condition followed the IOR improvised wet decontamination procedure and the interim decontamination procedure, as outlined above.

IORInitial Operational Response

**Table 2:** Mean (SD) and range for decontamination condition variables

	Control	Dry	Wet	Dry+interim	Wet+interim
Ambient	22.8 (3.7)	21.2 (2.6)	23.3 (2.5)	23.2 (3.1)	23.4 (3.1)
temperature (°C)	16.5−26.4	15.7–25.9	16.9–28.4	16.5–29.4	17.7–30.4
Quantity of white	–	10 (5.1)	–	10 (5.6)	
roll (sheets, n)		5–20		3–20	
Dry	–	2:37 (0.02)	–	2:39 (0:01)	
decontamination		1:49 – 3:03		1:59 – 3:03	
time (min:s)					
Water temperature—	–	–	23.8 (2.3)		25.4 (2.1)
improvised wet (°C)			20.7–28.3		21.6–30.1
Water temperature—	–			21.8 (2.7)	21.4 (1.8)
interim shower (°C)				16.2–26.8	18.2–23.9

### Patient and public involvement

None.

### Study procedure

Timelines for each stage of the study procedure in each decontamination condition are presented in online supplemental materials 1.

Participants were asked to avoid certain foods and consumer products containing MeS within 24 hours, and avoid showering within 2 hours, of their study session (see online supplemental materials 2). On arrival, participants’ adherence to prestudy instructions was assessed, along with checks for any changes in medical history that may impact their eligibility. Prior to study commencement, participants supplied a baseline urine sample (10–50 mL) in a 50 mL Falcon tube (Fisher Scientific, UK). The total volume of urine excreted was recorded. Urine samples were immediately stored at 4 °C.

Participants then changed into either black or dark blue polyester/nylon swimwear and black shoes provided. A baseline (presimulant application) UV image (UV1) was captured.

A simulant (1:1 v/v of MeS with vegetable oil, to which the fluorescent marker Invisible Red S was added, to a final concentration of 4 mg/mL) was applied to predetermined locations on the participants’ shoulder, forearm and calf chosen to represent hard, medium and easy areas to decontaminate, respectively (see online supplemental materials 3), as per Southworth *et al*.^7^ A simulant volume of 2 µL was applied to each analytical application site, resulting in an application of 1 µL (1.174 mg) of MeS per site. To facilitate simulant detection in urine, five parallel lines of 10×10 µL simulant were applied to the participant’s mid-back, 10×10 µL applications were applied to the upper arm in a single row and 10×10 µL applications were added to the lower leg in a triangular pattern (700 µL in total). The total application for systemic purposes equates to 350 µL (411 mg) of MeS.

Process controls were created by applying 2 µL of simulant onto two D-Squame discs. One disc was placed into 10 mL dichloromethane (DCM) immediately following simulant application to act as a control against theoretical recovery, while the other was placed into a vial of DCM during skin sample collection to normalise against potential evaporative loss of simulant due to ambient conditions.

Improvised decontamination conditions (conditions B–E) began at 15 min post simulant application to replicate the expected time for improvised decontamination initiation in IOR guidance.^11^ Interim decontamination conditions (conditions D and E) began at 25 min post simulant application, as this is the expected time at which an interim shower would be set up by FRS responders. During decontamination, although simulant was only applied to specific areas of the participant’s body, participants were asked to decontaminate as if they had been contaminated all over their body.

Following decontamination procedures, skin samples were taken from the participants application sites using tape strip sampling 30 min after simulant application, as per Southworth *et al*.^7^ Six tape strip discs were taken in total and placed in three prefilled vials containing 10 mL of DCM (vial A—discs 1 and 2, vial B—discs 3 and 4, vial C—discs 5 and 6). Samples were stored at 5 °C until subsampling. Aliquots (1 mL) were subsampled from each sample vial at least 24 hours following sample collection and were coded to blind the analyst of the samples.

Participants collected a sample every time they urinated for 8 hours following simulant application. For each sample, participants recorded the total volume urinated and collected 50 mL in a falcon tube (Fisher Scientific). Participants recorded the time of each urine sample and provided details of any missed samples. Sample aliquots (1.8 mL) were transferred by a researcher to a Cryo.S vial (Greiner Bio-One) and stored at −20°C prior to analysis.

### Skin and urine sample analysis

Skin sample analysis was conducted by gas chromatography tandem mass spectrometry (GC-MS/MS) as outlined by James *et al*^12^ and MeS was extracted from urine samples by supported liquid extraction prior to GC-MS/MS.^12^

### UV photography and image analysis

Whole-body UV images were captured at a total of six time points during the study session to record the spread and intensity of the simulant on the skin, as per Southworth *et al*.^7^

### Interpretation and statistics

Outcome measures were analysed using repeated measures analysis of variance, with decontamination condition as an independent variable. Planned contrasts compared the following: the four decontamination conditions compared with the no decontamination control; the main effect of the decontamination stage (improvised only compared with improvised plus interim conditions); the main effect of type of improvised decontamination (dry vs wet conditions); and the interaction between the decontamination stage and type of improvised decontamination. For skin samples and UV-illuminated image measures, the application site (arm, leg, shoulder) was also included as an independent variable to investigate differential effects of decontamination protocols for each application site. Planned contrasts compared the amount of simulant recovered on the shoulder to the arm and leg and compared the arm to the leg. For all outcome measures, Alpha was 0.05, with Huynh-Feldt sphericity corrections applied for repeated measures effects.^13^ Analyses were conducted using IBM SPSS Statistics V.25.

## Results

All participants completed all study conditions. Six participants’ data were removed from urine analysis due to missed samples. Process controls taken during the study showed no cross-contamination or major deviation in conditions between trial days.

### Skin sample analysis

MeS was detected above the limit of quantitation (0.23 ng/mL) in all skin samples, including the lower D-Squame disks. Statistical analysis showed a significant main effect of decontamination condition on MeS recovery, *F*(4,44)=7.69, p=0.017 (online supplemental materials 4A). Recovery of the simulant was significantly lower after decontamination compared with no decontamination control, *F*(1,11)=7.75, p=0.018. Mean total recoveries were 184.90 µg (SD 212.60) for controls (about 15% of the applied dose), and 12.76 µg (SD 6.87), 17.45 µg (SD 20.40), 9.23 µg (SD 4.90) and 9.27 µg (SD 4.53) for conditions B, C, D and E, respectively. Pairwise comparisons showed significantly lower recovery in all decontamination conditions compared with the control (all ps<0.05). Planned contrasts indicated no significant difference between improvised decontamination alone and combined improvised and interim decontamination, *F*(1,11)=2.59, nor between improvised dry and wet conditions, *F*(1,11)=0.88, or their interaction, *F*(1,11)=0.47.

Decontamination effectiveness varied by application site. Analysis showed a significant main effect of application site, *F*(2,22)=6.38, p=0.028, but no significant interaction between decontamination condition and site, *F*(6,66)=2.77. Recovery was significantly higher on the shoulder compared with the arm and leg, *F*(1,11)=6.40, p=0.028, indicating the shoulder was less effectively decontaminated. The difference in recovery between the shoulder and other sites was also significantly higher with improvised decontamination alone than with combined improvised and interim decontamination, *F*(1,11)=5.20, p=0.043. There was a trend towards significantly lower recovery on the shoulder with combined decontamination compared with improvised alone, *F*(1,11)=4.23, p=0.064, but no significant difference in recovery on the arm and leg between the two methods, *F*(1,11)=1.09. Thus, interim decontamination did not affect the recovery on the arm and leg but did reduce recovery on the shoulder. Recovery was consistent across the arm and leg in all decontamination conditions, *F*(1,11)=.70. In the control condition, there was no significant main effect of application site, *F*(2,22)=1.35, and no significant difference in recovery from the shoulder compared with the arm and leg, *F*(1,11)=1.17, indicating that recovery of simulant was consistent across application sites when no decontamination was conducted (table 3).

**Table 3:** Mean (SD) total amount of MeS (μg) recovered from skin for each application site in each decontamination condition

Application site	Sample vial	A—control	B—dry	C—wet	D—dry+interim	E—wet+interim
		(N=12)	(N=12)	(N=12)	(N=12)	(N=12)
Arm	Vial A	18.15 (20.59)	0.94 (0.80)	0.94 (0.52)	1.10 (0.54)	1.14 (0.52)
	Vial B	1.22 (1.50)	0.77 (0.61)	0.76 (0.44)	0.98 (0.49)	0.87 (0.43)
	Vial C	1.02 (1.03)	0.76 (0.70)	0.72 (0.40)	0.79 (0.46)	0.91 (0.53)
	**Total**	**20.39(20.60)**	**2.47(2.01)**	**2.42(1.31)**	**2.87(1.40)**	**2.91(1.37)**
Leg	Vial A	63.94 (118.35)	0.91 (0.62)	0.91 (0.62)	0.94 (0.73)	0.99 (0.51)
	Vial B	2.73 (2.98)	0.71 (0.56)	0.84 (0.34)	0.87 (0.57)	0.90 (0.57)
	Vial C	1.70 (1.79)	0.59 (0.42)	0.78 (0.45)	0.93 (0.67)	0.88 (0.50)
	**Total**	**68.37(121.14)**	**2.21(1.47)**	**2.54(1.25)**	**2.75(1.92)**	**2.77(1.49)**
Shoulder	Vial A	89.39 (152.87)	5.32 (5.27)	9.55 (17.15)	1.81 (1.23)	1.49 (0.76)
	Vial B	5.15 (8.58)	1.16 (0.84)	1.91 (2.17)	0.92 (0.60)	1.04 (0.56)
	Vial C	1.60 (0.83)	1.60 (2.34)	1.04 (0.48)	0.89 (0.46)	1.06 (0.76)
	**Total**	**96.14(161.40)**	**8.08(5.90)**	**12.50(19.33)**	**3.62(2.05)**	**3.59(1.87)**
Total		**184.90(212.60)**	**12.76(6.87)**	**17.45(20.40)**	**9.23(4.90)**	**9.27(4.53)**

### Correlations between dry decontamination variables and decontamination efficacy

The number of pieces of white roll used by each participant during dry decontamination ranged between 3 and 20. There was no significant correlation between number of pieces of white roll used and total MeS recovered within either dry decontamination condition, r_s_=−0.07, r_s_=−0.16.

Time spent on dry decontamination ranged between 1 min 9 s and 3 min. There was no significant correlation between time spent on dry decontamination and total MeS recovered within either dry decontamination only condition, r_s_=−0.09, r_s_=0.28, for dry and interim combined condition.

### Urine sample analysis

There was no significant main effect of decontamination condition on MeS excreted in urine, *F*(4,20)=0.82, and no significant difference between the decontamination conditions and the no decontamination control, *F*(1,5)=2.44, (online supplemental materials 4B).

### UV image analysis

All whole-body UV images yielded data pertaining to simulant area and emittance. For postapplication images (UV2), there was no significant difference between the decontamination conditions in either simulant area, *F*(4,44)=1.57 or emittance, *F*(4,44)=1.57, demonstrating that prior to decontamination both area and emittance were consistent across the decontamination conditions.

For postdecontamination images (UV5), there was a significant main effect of decontamination condition for both area, *F*(4,44)=13.61, p=0.001 (online supplemental materials 5A), and emittance, *F*(4,44)=9.49, p=0.003 (online supplemental materials 5B). Area and emittance were both significantly lower following decontamination compared with no decontamination control, *F*(1,11)=12.91, p=0.004 and *F*(1,11)=8.96, p=0.012, respectively. Pairwise comparisons confirmed that both were significantly lower in each of the decontamination conditions compared with control, except for the improvised dry only condition (p=0.084 for area; p=0.149 for emittance; all others p<0.01). Planned contrasts also found that area and emittance were significantly lower following combined improvised and interim compared with improvised only decontamination, *F*(1,11)=41.18, p<0.001 and *F*(1,11)=31.51, p<0.001 respectively, and in the wet compared with dry conditions, *F*(1,11)=10.34, p=0.008 and *F*(1,11)=6.64, p=0.026, respectively. This difference in area and emittance between the dry and wet conditions was significantly reduced by the inclusion of interim decontamination, *F*(1,11)=7.29, p=0.021 and *F*(1,11)=5.48, p=0.039, respectively.

Across the active decontamination conditions, for UV5 images, there was a significant main effect of application site for both area and emittance, *F*(2,22)=13.57, p=0.001 and *F*(2,22)=13.18, p=0.001, respectively. There was also a significant interaction between application site and decontamination condition, *F*(6,66)=3.68, p=0.035 and *F*(6,66)=4.38, p=0.029, respectively. Planned contrasts found that area and emittance were significantly higher on the shoulder compared with the arm and leg, *F*(1,11)=19.67, p=0.001 and *F*(1,11)=15.60, p=0.002, respectively, and this difference was significantly higher in the improvised only compared with improvised plus interim conditions, *F*(1,11)=20.04, p=0.001 and *F*(1,11)=17.34, p=0.002, respectively. Subsequent analyses found that while area and emittance were significantly higher on the shoulder compared with the arm and leg in the improvised only conditions, *F*(1,11)=21.07, p=0.001 and *F*(1,11)=16.24, p=0.002, respectively, there was no significant difference in area or emittance for the shoulder compared with the arm and leg in the interim conditions, *F*(1,11)=2.51 and *F*(1,11)=2.63, respectively. There was no significant difference in area or emittance between the arm and leg across the active decontamination conditions, *F*(1,11)=0.13, and *F*(1,11)=0.65, respectively. Within the control condition, there was no significant main effect of application site on either area or emittance, *F*(2,22)=0.40 and *F*(2,22)=1.12, respectively, and no significant difference in area or emittance on the shoulder compared with the arm and leg, *F*(1,11)=1.48, and *F*(1,11)=2.11, respectively (tables4  5).

**Table 4:** Mean (SD) area of fluorescence (cm^2^) for each application site in each decontamination condition

		Decontamination condition				
Image	Site	A—control (n=12)	B—dry (n=12)	C—wet (n=12)	D—dry+interim (n=12)	E—wet+interim (n=12)
UV2	Arm	3.55 (3.07)	4.59 (2.22)	5.08 (3.34)	4.42 (2.55)	4.59 (3.09)
	Leg	2.58 (1.23)	3.10 (1.44)	2.93 (1.30)	2.19 (0.83)	2.56 (1.29)
	Shoulder	3.23 (1.91)	4.66 (1.95)	3.69 (1.39)	3.68 (1.71)	3.42 (1.94)
	**Total**	**9.36(5.70)**	**12.35(3.84)**	**11.70(5.27)**	**10.28(4.13)**	**10.56(5.89)**
UV3	Arm	4.00 (3.60)	1.82 (2.09)	0.22 (0.40)	1.54 (2.01)	0.35 (0.53)
	Leg	3.62 (2.05)	1.09 (1.15)	0.38 (0.83)	0.96 (0.91)	0.19 (0.37)
	Shoulder	3.87 (2.78)	3.84 (2.51)	1.54 (1.90)	2.26 (2.00)	1.11 (1.11)
	**Total**	**11.49(7.60)**	**6.74(3.13)**	**2.15(2.15)**	**4.76(3.45)**	**1.65(1.62)**
UV4	Arm	3.59 (3.40)	1.63 (2.00)	0.13 (0.29)	1.25 (1.66)	0.34 (0.53)
	Leg	3.64 (2.12)	0.98 (1.01)	0.31 (0.69)	0.81 (0.80)	0.11 (0.33)
	Shoulder	3.98 (3.00)	3.73 (2.55)	1.51 (1.79)	2.23 (2.02)	1.06 (1.10)
	**Total**	**11.21(7.64)**	**6.34(3.13)**	**1.94(1.95)**	**4.29(2.75)**	**1.51(1.60)**
UV5	Arm	3.33 (3.54)	1.27 (1.76)	0.14 (0.39)	0.19 (0.40)	0.12 (0.27)
	Leg	3.49 (2.17)	0.88 (0.88)	0.31 (0.70)	0.22 (0.36)	0.05 (0.17)
	Shoulder	3.92 (3.16)	3.52 (2.64)	1.50 (1.88)	0.44 (0.60)	0.20 (0.35)
	**Total**	**10.74(8.05)**	**5.67(3.31)**	**1.95(2.11)**	**0.85(0.83)**	**0.37(0.65)**
UV6	Arm	2.34 (2.70)	0.76 (1.13)	0.06 (0.15)	0.10 (0.21)	0.08 (0.18)
	Leg	2.27 (1.66)	0.52 (0.57)	0.23 (0.57)	0.16 (0.33)	0.03 (0.09)
	Shoulder	2.47 (2.05)	2.27 (2.14)	0.84 (1.12)	0.28 (0.30)	0.14 (0.27)
	**Total**	**7.08(5.87)**	**3.55(2.37)**	**1.13(1.30)**	**0.54(0.49)**	**0.49(0.62)**

**Table 5:** Mean (SD) fluorescent emittance index for each application site in each decontamination condition

		Decontamination condition				
Image	Site	A—control (n=12)	B—dry (n=12)	C—wet (n=12)	D—dry+interim (n=12)	E—wet+interim (n=12)
UV2	Arm	3.01 (2.83)	3.84 (2.14)	4.71 (3.06)	3.88 (1.82)	4.55 (3.13)
	Leg	1.93 (0.93)	2.32 (1.05)	2.21 (0.83)	1.75 (0.61)	2.20 (0.91)
	Shoulder	2.68 (1.79)	4.31 (2.40)	3.49 (1.79)	3.51 (1.77)	3.16 (1.47)
	**Total**	**7.62(5.06)**	**10.47(4.02)**	**10.41(4.80)**	**9.14(3.25)**	**9.90(5.02)**
UV3	Arm	3.21 (3.10)	1.21 (1.58)	0.13 (0.26)	0.85 (1.04)	0.23 (0.36)
	Leg	2.24 (1.33)	0.55 (0.62)	0.17 (0.37)	0.52 (0.61)	0.09 (0.17)
	Shoulder	3.15 (2.68)	3.18 (2.65)	1.28 (1.73)	1.82 (1.89)	0.98 (1.20)
	**Total**	**8.60(6.32)**	**4.93(3.18)**	**1.58(1.73)**	**3.20(2.60)**	**1.30(1.45)**
UV4	Arm	2.73 (2.96)	1.04 (1.40)	0.08 (0.19)	0.70 (0.88)	0.22 (0.36)
	Leg	2.23 (1.42)	0.48 (0.54)	0.13 (0.29)	0.47 (0.64)	0.05 (0.16)
	Shoulder	3.19 (2.91)	3.06 (2.65)	1.29 (1.76)	1.77 (1.85)	1.00 (1.25)
	**Total**	**8.14(6.52)**	**4.58(3.09)**	**1.50(1.74)**	**2.95(2.26)**	**1.27(1.50)**
UV5	Arm	2.61 (3.24)	0.83 (1.24)	0.07 (0.21)	0.10 (0.20)	0.07 (0.15)
	Leg	2.14 (1.50)	0.43 (0.46)	0.13 (0.28)	0.13 (0.28)	0.02 (0.07)
	Shoulder	3.15 (3.11)	2.93 (2.82)	1.17 (1.59)	0.23 (0.31)	0.14 (0.25)
	**Total**	**7.90(7.15)**	**4.19(3.28)**	**1.37(1.60)**	**0.45(0.49)**	**0.22(0.38)**
UV6	Arm	1.89 (2.51)	0.48 (0.79)	0.03 (0.07)	0.06 (0.11)	0.05 (0.11)
	Leg	1.42 (1.27)	0.25 (0.29)	0.09 (0.23)	0.09 (0.23)	0.01 (0.03)
	Shoulder	2.09 (2.22)	1.96 (2.03)	0.65 (1.00)	0.15 (0.17)	0.10 (0.21)
	**Total**	**5.41(5.57)**	**2.69(2.22)**	**0.77(1.01)**	**0.29(0.32)**	**0.32(0.39)**

## Discussion

This study evaluated the effectiveness of improvised and interim decontamination methods, both alone and in sequence. Our results align with previous research, showing significantly lower skin contaminant levels after decontamination compared with controls.^7^ This supports current IOR guidance which recommends improvised decontamination following chemical exposure until more structured interventions, such as interim or SOR units, are available.^10^

Building on previous research, our study found that combining improvised and interim decontamination reduced simulant levels on harder-to-reach areas, such as the shoulder, more effectively than improvised methods alone. This highlights the importance of performing interim decontamination after chemical exposure.

Urinalysis results showed no difference in MeS excretion levels between the conditions. However, low sample size for the urine analysis reduced statistical power, making it harder to detect potential differences. Additionally, MeS’s volatility suggests that skin absorption might not be the only route; respiratory exposure could also play a role. A study on chemical warfare agents, including MeS, found vapour levels occasionally exceeded safe thresholds after decontamination, indicating respiratory exposure risk.^14^ More research on respiratory exposure is needed before drawing definitive conclusions about vapour exposure risks.

Using vegetable oil as a ‘carrier’ likely affected MeS’s physicochemical properties, increasing lipophilicity and potentially reducing the efficacy of wet decontamination. Despite this limitation, the oil was necessary to maintain detectable MeS throughout the study. This approach provided insights into strategies for MeS and similar chemicals, but generalising to other chemicals with different properties is challenging.

Participants in this study carried out decontamination under direct researcher guidance. In real incidents, casualties often outnumber responders, making communication crucial for effective decontamination.^15^ Thus, this study might overestimate effectiveness since participants received clear, one-on-one instructions.

For improvised wet decontamination, participants used buckets of water with identical volumes. However, in real incidents with several casualties, limited water sources may lead to uneven distribution and less effective decontamination. This further highlights the need for interim decontamination after improvised decontamination.

Managing contaminated water run-off is also crucial. Removing clothing can reduce the amount of chemical in water run-off,^16^ but the Environment Agency and local sewage companies should be notified if containment is inadequate. Despite this, the priority for emergency services is to save life and reduce harm, so concerns about run-off should not delay urgent decontamination.^16^

Exploring a wider range of safe chemicals with different physicochemical properties could improve universal decontamination protocols. James *et al* identified benzyl salicylate as a suitable simulant for persistent, lipophilic compounds,^17^ and it has been used in recent studies of hair and specialist decontamination.^6 18^ Future research should test emergency decontamination procedures with this simulant on human skin.

This study focused solely on IOR, but in real incidents IOR is usually followed by SOR with mass decontamination units. Understanding the additional benefits of SOR is crucial, as it often follows IOR. Collins *et al*^18^ found SOR provided additional benefits for removing the more persistent benzyl salicylate, but not for MeS. This suggests that for chemicals not effectively removed by improvised and interim methods, adding SOR is likely to be beneficial.

In conclusion, this study reinforces the importance of decontamination following chemical exposure. While evidence for the overall benefits of using both improvised and interim decontamination sequentially was partial, results consistently showed that improvised methods alone were less effective for hard-to-reach areas. Using interim decontamination after improvised methods improved effectiveness in these areas. This study supports a two-step approach where emergency responders start with improvised decontamination and then use interim decontamination as additional resources become available, ensuring the most effective on-scene decontamination.

## supplementary material

10.1136/emermed-2024-214221 online supplemental file 1

10.1136/emermed-2024-214221 online supplemental file 2

10.1136/emermed-2024-214221 online supplemental file 3

10.1136/emermed-2024-214221 online supplemental file 4

10.1136/emermed-2024-214221 online supplemental file 5

## Data Availability

Data are available on reasonable request.
