# Supplementary figures and images for: Mass casualty decontamination following a chemical incident: evaluating improvised and interim decontamination protocols in a controlled cross-over volunteer study
Source: Emerg Med J. 2024 Dec 4;42(1):e214221. doi: 10.1136/emermed-2024-214221 (PMC11874368; doi:10.1136/emermed-2024-214221)

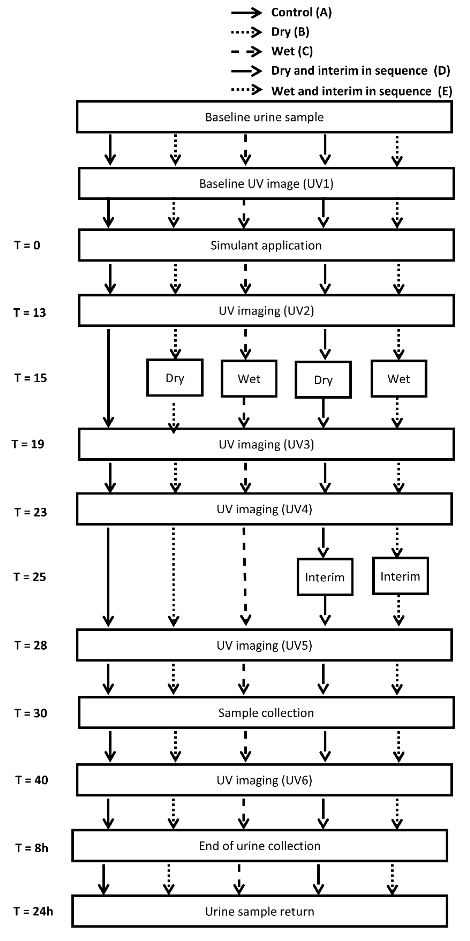

Supplement: online supplemental file 1 [file emermed-42-1-s001.PNG]

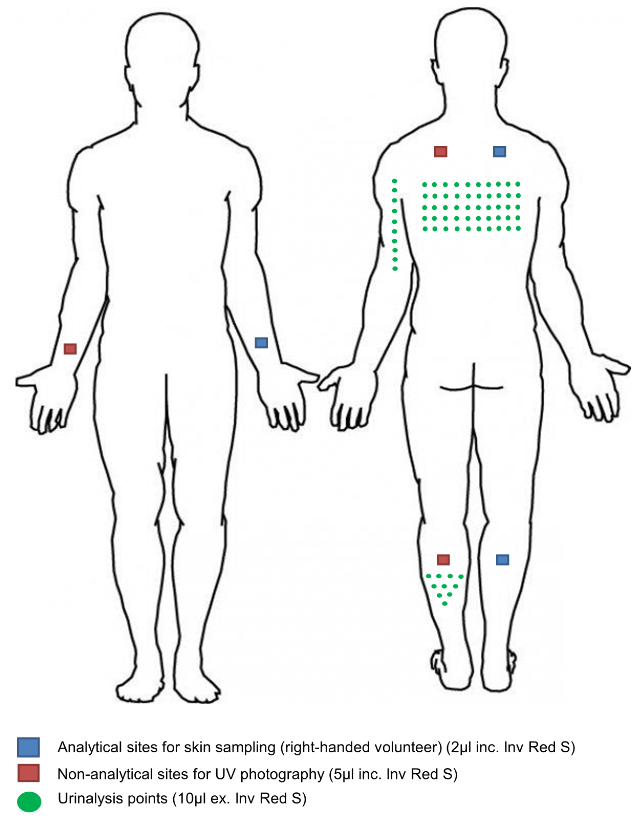

Supplement: online supplemental file 3 [file emermed-42-1-s003.PNG]

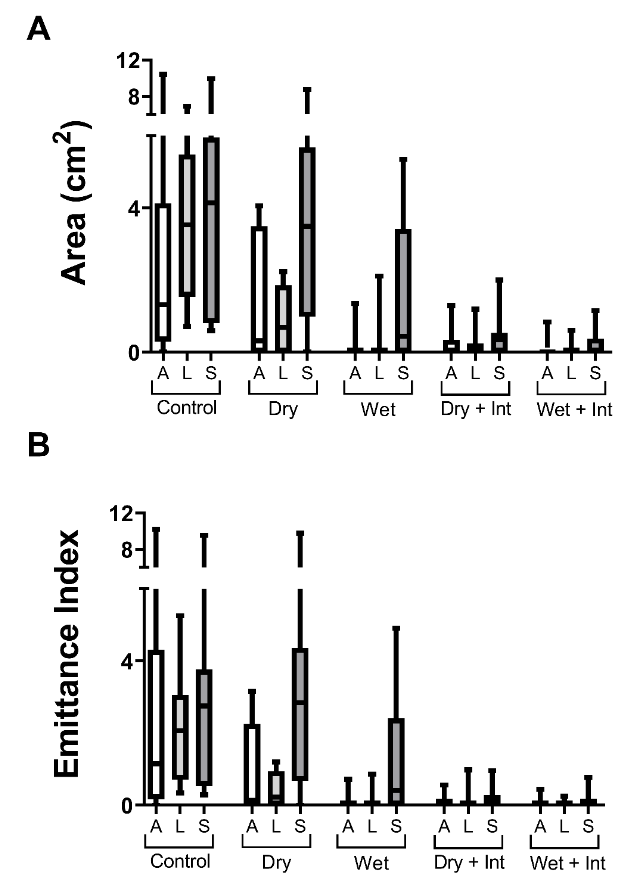

Supplement: online supplemental file 4 [file emermed-42-1-s004.PNG]

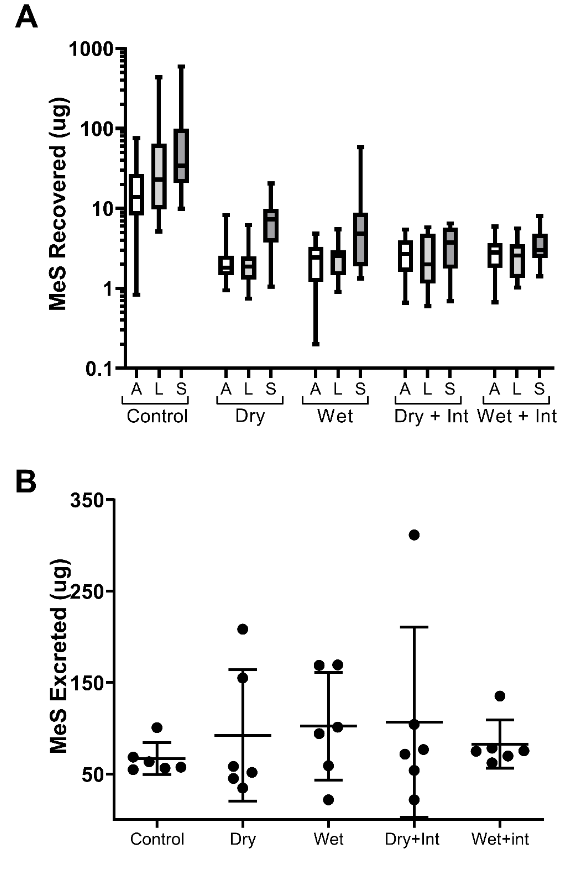

Supplement: online supplemental file 5 [file emermed-42-1-s005.PNG]
