# Supplementary material for: Mass casualty decontamination following a chemical incident: evaluating improvised and interim decontamination protocols in a controlled cross-over volunteer study
Source: Emerg Med J. 2024 Dec 4;42(1):e214221. doi: 10.1136/emermed-2024-214221 (PMC11874368; doi:10.1136/emermed-2024-214221)
Supplement: online supplemental file 2 [file emermed-42-1-s002.pdf]

Supplementary Materials 2

Food and consumer products containing MeS that volunteers were instructed not to use or consume in the 24 hours before the study session

The day before the study day (and the day after):

- Please **do not consume any alcohol** in the **24 hours before and 8 hours after** the day of the study.
- Please **do not consume any aspirin** or apply any products containing aspirin in the **24 hours before and 24 hours after** the day of the study, including muscular rubs or ointment.
- **Do not consume or use any products containing methyl and other salicylates** the **24 hours before and 8 hours after** the day of the study (see Appendix A for a complete list of foods and consumer products that should be avoided).

The morning of the study:

- Please do not apply any deodorant or shower gel the morning before the study.
- Please finish showering/washing **at least 2 hours** before the commencement of the study.
- Please remove any body piercings.
- For the first study session, please wear loose-fitting clothing so we can access the top of your arm to measure your blood pressure.

During and after the study:

- Please be aware that the researcher may need to remove body hair from small areas approximately 2-3cm^2 on the back of the legs, arms and back.
- Please do not shower or bath for **four hours** after you have left the trial site.
- Please refrain from intercourse in the **24 hours** following study completion.

Appendix A: List of foods and products to be avoided in the 24 hours before and 8 hours after the study

**MUST be avoided:**	**SHOULD be avoided where possible:**
Peppers	Courgettes
Tomatoes (including sauce)	Mushrooms
Grapes	Apricots
Oranges (including juice)	
Strawberries	

Mints and peppermints	
Chewing gum	
Tea and coffee	
Mouthwash	
Acne products	
Muscle and joint pain cream (e.g. Deep Heat)	
Skin cleaners and exfoliants	
Perfumed products (e.g., cologne, moisturiser)	
Root beer	
Alcohol	
